# Improved localization of language areas using single voxel signal analysis of unprocessed fMRI data

**DOI:** 10.3389/fradi.2022.997330

**Published:** 2022-09-22

**Authors:** Leonard Fetscher, Marion Batra, Uwe Klose

**Affiliations:** Department of Diagnostic and Interventional Neuroradiology, University Clinic of Tuebingen, Radiological Clinic, Tuebingen, Germany

**Keywords:** task-based fMRI, hemodynamic response function (HRF), language fMRI, *t*-Map, filter, HRF-dependent parameters, unprocessed fMRI

## Abstract

Activated brain regions can be visualized and localized with the use of fMRI (functional magnetic imaging). This is based on changes in the blood flow in activated regions, or more precisely on the hemodynamic response function (HRF) and the Blood-Oxygen-Level-Dependent (BOLD) effect. This study used a task-based fMRI examination with language paradigms in order to stimulate the language areas. The measured fMRI data are frequently altered by different preprocessing steps for the analysis and the display of activations. These changes can lead to discrepancies between the displayed and the truly measured location of the activations. Simple *t*-maps were created with unprocessed fMRI data, to provide a more realistic representation of the language areas. HRF-dependent single-voxel fMRI signal analysis was performed to improve the analyzability of these activation maps.

## Introduction

Language fMRI (functional magnetic resonance imaging) is widely used in pre-operative diagnostics to localize the language areas, mainly Broca's and Wernicke's areas (1–4). An accurate localization of the variably located language areas (LAs) is important, as injury even to only small parts of the language critical regions can cause aphasia (1, 3–6). Aphasia is a severe disease in which the patient, depending on the manifestation, can be completely deprived of the ability to communicate with other people or to understand them. This is accompanied by a reduced quality of life, depression, and reduced life expectancy (7). In contrast to the preservation of language areas stands the importance of the resection of the tumor. The preservation of too much tissue can result in an insufficient resection of the tumor, leading to a potential reoccurrence of the tumor and affecting the radiochemical treatment, the outcome, and the survival negatively (8–10). The precise localization of the (functionally important) language areas has a direct impact on neurosurgical treatment, especially in the case of lesions close to the LA. Language fMRI is widely used, for the localization of functional brain areas, offering a quick-to-perform, accessible, and non-invasive option for brain mapping. However, the utility of fMRI is questioned in the literature, since the sensitivity and specificity of this method vary greatly in different studies (11). The data are sometimes too imprecise to delineate the exact risk-free area for lesions that lie close to the functional areas. These inaccuracies are partly due to the cooperation of the patient or the limited resolution of the fMRI technique itself but can also be caused by the analysis of the fMRI data and certain preprocessing steps. The analysis of fMRI data in block design is based on statistical differences in the hemodynamic response function (HRF) between the activation block and the rest block. The differences are calculated using, e.g., a *t*-test or a general linear model (GLM) to identify activations. Several processing steps, such as a motion correction or smoothing, are performed in a clinical standard protocol, even though the modification of statistical analysis and the processing of the data can lead to a decrease in significance and an increase in evaluation errors (12, 13). Especially smoothing the data can change the actual location and the exact boundaries of the measured activations, making a reliable evaluation of the map and the separation of merging activations difficult.

Various aspects must be taken into consideration when evaluating fMRI data. The clinician must decide whether the shown activation is an artifact, an unwanted activation of another functional region (e.g., visual cortex), or a true language activation. Additionally, the spatial extent must be estimated after the targeted regions were identified. An activation map only shows a probable location and extent of activated regions (14), already making the displayed boundaries uncertain. Accordingly, a realistic display of the activations is of great importance, as it directly affects the validity of the evaluation and thereby the outcome of the patients.

This study focused on displaying the truly measured location of activations by using unprocessed block design fMRI data for the creation of a simple *t*-map. HRF-based time courses (TCs) of different regions and artifacts were created and closely analyzed to define the characteristics of the HRF that indicate language activations. The analysis was performed on task-dependent block-design fMRI data. A certain shape, such as a higher signal following the start of the activation block, was expected in the HRF of the language areas based on this task-based approach (12). There are subject- (15) as well as region-dependent differences in HRF (14, 16–18) that were used to define a language area-specific response to the stimuli. Previous findings of the Blood-Oxygen-Level-dependent (BOLD) effect (19), and common characteristics of the HRF, such as time to peak, height and width of the activation (12, 16, 19), were incorporated. The combined information about the HRF shape of the TCs of activated voxels in the LAs was then used to create a filter that identifies language voxel by voxel and separates those from artifacts.

## Materials and methods

### Data

In this study, two different sets of fMRI data were used, one of patients and one of healthy subjects. The data of the healthy subjects were obtained through a prospective study conducted by us. The subjects (*n* = 8) underwent 3 language fMRI scans, each with language paradigms using a visual stimulus presentation (24 scans in total). The scans for this study were performed in the University Hospital of Tuebingen. The study was approved by the Ethics Committee of the University of Tuebingen. Additionally, prior to the testing, all subjects gave their written consent to participate in this study and to be informed in the event of a pathological incidental finding.

The dataset of patients, which was used as a patient control group, contained the fMRI data of 28 patients. All patients had to undergo an fMRI examination due to clinical indications, while being treated in the University Hospital of Tuebingen. All patients gave written consent for these MRI data to be use for scientific purposes. As with the subjects, language paradigms with visual stimulus presentation were also used here. The data were provided in anonymous form, without further patient information. Using this dataset, the performance of the filtered *t*-map was evaluated, and the clinical suitability was tested.

### MRI-Sequence

All scans were performed with two Siemens 3 T whole-body scanner (model: MAGNETOM Skyra and MAGNETOM Prisma, Siemens, Germany). For the functional imaging, an echo planar imaging sequence (EPI; T2*-weighted, 104 volumes, slice thickness 3.0 mm, matrix 96, FV 245 mm × 245 mm, gap 0.75 mm, TR 3 s, TE 36 ms) was used.

### Paradigms

The task-dependent fMRI scans were performed using different language paradigms (paradigm size 16) in a block design. The duration of one block was 24 s containing eight volumes. The examination consisted of two blocks performed alternately, an activation block and a rest block, starting and ending with a rest block. There were 6 language and 7 rest blocks in total. In the subject study, three language paradigms were used for the activation block: verb-generation, naming and sentence completion, totaling 3 paradigms per subject. During the rest block, the subject was instructed to repeatedly form and open a fist with both hands. This action was prompted visually with the word “fist”.

The examination of the patients was the same as the subject study, with 6 activation and 7 rest blocks. The language paradigm used here was the verb generation task. The rest block also consisted of an activated pause, consisting of the fist closure that was prompted visually with the word “pause”.

The visual presentation of the tasks was in written form viewable by the subject/patient on a monitor *via* a mirror attached to the head coil.

### Creation of activation maps

In this study, two different techniques were used to create the activation maps. For both techniques, only the functional MRI data, which were acquired using the EPI-Sequence, was used to identify and display the activations; therefore, no co-registration was needed. Since the maps were created using only the functional MRI data, the activations were displayed in detail, but the anatomical mask was of low quality and the maps consisted of a limited number of slices. Further preprocessing steps, which are mainly used in neuroscience, but have little to no relevance in clinical routine, such as slice-timing or normalization, were also not used for this study. This avoided possible errors or changes that could be caused by the preprocessing of the data and kept the displayed activations similar to the originally measured data.

One technique for the activation maps was the creation of a GLM map. The activations were defined through the calculation of the functional fMRI data with the “Inline BOLD Imaging” included in the syngo.MR software of SIEMENS Healthineers, with “Inline calculation of *t*-statistics (*t*-maps) based on a GLM including the hemodynamic response function and correcting for slow drift” (Siemens Medical Solutions USA). Through creating these GLM maps, the fMRI data were pre-processed with motion correction, a spatial filter with a filter width of 5.0 mm (“smoothing”), a temp. highpass filter and the modeling of transition states. The paradigm size was 16, but the first time point (TP) of each block (TP 1 and 9) was ignored. In the following, these maps are referred to as GLM maps. The threshold of the GLM maps was set to *t* = 2.2, as this was the *t*-limit chosen for the *t*-maps.

The second technique that was used for the creation of activation maps was a simple *t*-map, on which the filtered *t*-map was created in the process. The special feature of these *t*-maps is that, in addition to co-registration and normalization, the pre-processing steps motion correction and smoothing were omitted, i.e., raw measurement data were used.

The *t*-maps were created with a Matlab (MATLAB 12.0, The MathWorks, Inc., Natick, Massachusetts, United States)-based computer program. The activations were calculated by comparing the TC signal of a voxel during the activation block to its TC during the rest block. The TC of each block was averaged over all 6 activation/rest blocks contained in one measurement. The first TP of each block (TP 1 and TP 9) was ignored. Significant TC differences between the activation and the rest block suggested a task-dependent activation. The differentiation between activation and no activation is based on the (in this study generally used) *t*-limit of *t* = 2.2. To define this *t*-limit, 48 fMRI data sets conducted by the subject study were used. The differences in the number of excluded activations at different *t*-limits were compared between activations in the LAs and all activations on the entire *t*-map. The *t*-limit of 2.2 was used for all maps, as it showed the best compromise between preserving language activations and deleting artifacts and unwanted activations.

### Calculation of the TCs and creation of time courses

The TCs were calculated in a way that made the comparison between the voxels and the different measurements possible. To observe positive and negative fluctuations in the BOLD-Signal, originating from changes in the blood flow caused by the HRF in activated regions, the mean of the signal strength (*y*-value) was defined as the reference of the TC. To compare the signal deflections, or the signal strength, a relative value was used instead of an absolute one. For the standardization of the signal strength (*y*-value), the TCs were converted to percent:


TC[%]=((TC−mean(TC)))/(mean(TC))×100

**Figure 1:**
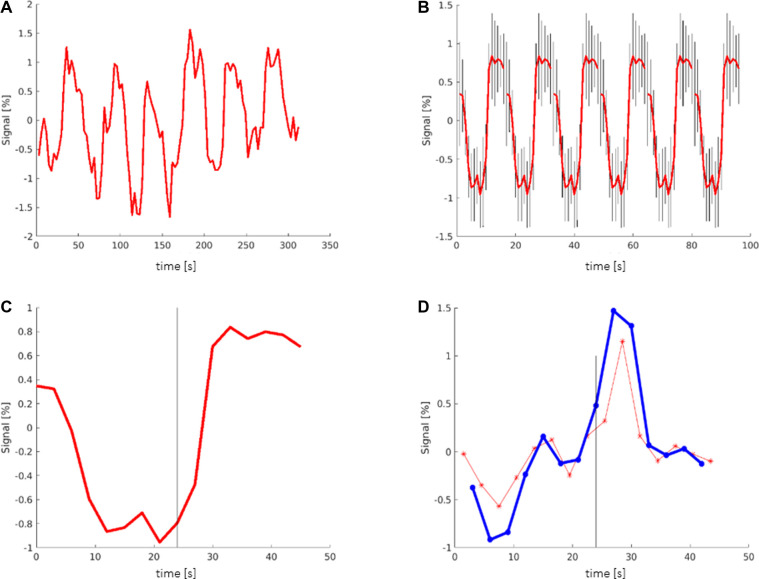
Creation of time courses: (**A**) TC (time course) showing the averaged signal of all voxels in the selected region (here: Broca's area). The *y*-axis shows the deviation of the signal from the mean value. (**B**) TC averaged over all 6 periods, including the maximum deviation (black bars) of the signal. (**C**) TC showing one period that contains the mean information of all 6 periods; black bar signals the start of the activation block at 24 s. (**D**) Red graph showing the derivation of the TC representing the slope (referring to the next time point (TP)). Blue graph showing the derivation of the TC representing the slope (referring to the second TP); black bar: start of the activation block at 24 s.

To create the TCs, regions of interest were selected, and the average TCs of all voxels contained in the selected region were plotted (Figure 1A). The TCs were then averaged over all 6 periods and plotted with the indication of the maximum deviation of the signal of each TP (Figure 1B) or summarized as one period (Figure 1C).

To examine the slope of the TCs, a graph of the derivation of the TC was created (Figure 1D). The slope of the TC was measured in two different ways. In one graph, the slope was calculated between one TP and its next TP, representing a period of 3 s (Figure 1D red). In the other graph, the slope of the TC was calculated between one TP and its second TP (Figure 1D blue). Ultimately, only the second graph was used, which showed more consistent values.

### Analysis of time courses and definition of parameters

Initially, activations of six different frequently activated regions were selected and saved as ROIs (regions of interest). The most important ROIs were the language areas (LAs), consisting of Broca's area and Wernicke's area. Furthermore, the visual cortex, which is activated by the presentation of the language paradigms, and the motor cortex, which is activated by language production, were saved as ROIs.

Additionally, two frequent locations of artifacts were saved as ROIs to observe the TCs of apparent activations that are not necessarily related to a functional region, the language paradigms or language production: The “ventricular artifact” includes apparent activations located on the border between the brain tissue and the ventricular system. These activations are evoked by small head movements, which can lead to a voxel changing its location from the brain tissue to intraventricular tissue and vice versa. This has an impact on the TC of this voxel and can make it look like a significant activation. The second artifact, the “frontal artifact”, is a collection of apparent activations located in the rostral region of the scan, including activations inside and outside the brain tissue (such as frontal, lobe, n. opticus, and blood vessels).

In the next step, the ROIs were grouped based on region across different scans and the averaged TC of each region was plotted.

To obtain the region-specific distribution of values, to observe interregional differences and to find HRF-dependent parameters, these averaged TCs were then compared to each other. This allowed the distinguishing between language activations and activations that lie outside the LAs or artifacts.

Appropriate limits had to be set to be able to implement these LA-specific parameters to the filter. A single-voxel TC analysis, calculating the value of each parameter (max./min signal strength, max./min. slope and timing of the max./min. slope) from the HRF of each individual voxel displayed as an activation on the *t*-map, was performed. The values of all activated voxels on the *t*-map were compared to the values of the voxels located in the LAs. The results were plotted as histograms and the differences between the values of both groups of voxels were examined to define the limits.

### Filter *t*-maps

To integrate the findings from the TC analysis in the creation of activation maps, a Matlab-based computer program working as a filter was designed. A single-voxel TC analysis of every voxel marked as activation on the *t*-maps was performed using the program, comparing the values of each TC to the limits of the parameters that were defined by the single voxel TC analysis.

Voxels with TCs not matching the limits of the parameters were marked as false activation and removed from the *t*-map. The parameters were initially applied individually to the *t*-maps to verify the limits and to add minor adjustments to the limits, representing the TCs of the language areas and minimizing the number of false-negative voxels. The main focus was on not excluding language activations, so the range of values was extended in some cases of uncertainty. After finalizing the limits for each parameter, all parameters were combined and applied to the *t*-map to create the final result, the filtered *t*-map.

### Evaluation of activation maps

Three aspects were included in the evaluation of the activation maps.

First, the general usability of the activation map was assessed by checking if both (clinically) relevant language areas, Broca's and Wernicke's areas, were displayed. The second aspect focused on the display and evaluability of the LAs. It was examined whether the language areas merge into directly adjacent activations or whether they are free-standing, making a spatially more precise identification possible. The third aspect assessed the general quality of the activation map. For this purpose, the ratio between the number of activated voxels in the LAs and the number of activated voxels outside the LAs, consisting of artifacts and unintentional activated voxels of functional areas co-activated by the stimuli, was calculated.

**Figure 2:**
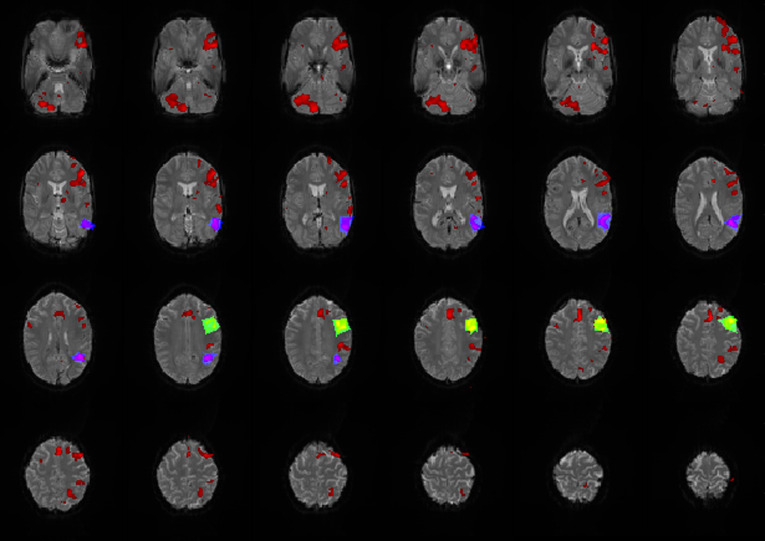
Exemplary map with broca's (green/yellow), wernicke's areas (blue/purple) and additional activations (red) marked. All activations that lay within the marked areas were attributed to the corresponding LA and were included as language activation in the calculation of the quotient.

An estimated outline of the LAs was drawn manually, based on anatomical knowledge of Broca's and Wernicke's area (Brodmann area 44,45 and 22) and the individual brain structure of the patient/subject, to count the number of activated voxels belonging to the LAs. It was not intended to represent the exact location of the LAs but is intended to include the entire expected area of the LAs so that all activated voxels located there and possibly belonging to the LAs could be counted (Figure 2). The number of activated voxels inside Broca's and Wernicke's areas was then divided by the total number of activated voxels on the whole map. The quotient represented the ratio of relevant language activations to additional activations and artifacts on the map. A larger quotient indicated the existence of less artifacts and/or more language activation, conclusively indicating a superior map.

This ratio was calculated for the GLM maps and the filter *t*-maps. To put this ratio into proportion, different cluster limits [deleting voxel clusters that consisted of less voxels than the limit (allowed cluster sizes of 1, 3, 4, 5, 9, and 13)], as well as different thresholds/*t*-limits (*t* = 1.5; 2.0; 2.2; 2.5), were applied to both maps. Data were only included in the calculation of the quotient if there was a language activation displayed on the maps. Activation maps in which the entire language activation was excluded, e.g., by increasing the cluster limit, were not included in the calculation, and were documented as excluded.

## Results

All scans performed during the prospective study with the subjects were successfully completed, with a good display of the activations and an acceptable number of artifacts, e.g., due to the movement of the subjects. From the patient data group, 10 patients had to be excluded due to incomplete data sets, poor signal, or excessive motion artifacts (based on the motion corrected GLM maps). No data set that showed bad quality only in the filter *t*-map (without motion correction) but good quality on the GLM map (with motion correction), was excluded. In total, the data of all 8 subjects (24 scans) and the data of 20 patients (28 scans) were used.

The *t*-maps were of adequate quality, even though no preprocessing was used. Additionally, the absence of motion correction did not have a major impact on the *t*-maps, as the subjects and patients were well positioned during the measurements, which proved to be sufficient for the limited spatial resolution of the fMRI data.

### *t*-limit

The differences in the number of excluded activations at different *t*-limits were compared between activations in the LAs and all activations on the entire *t*-map. Language activations were still displayed on the map at higher *t*-values, while many of the non-language activations were excluded. At the *t*-value of 2.2, 80% of the activated voxels of the entire map were excluded, while only 55% of the language activations were excluded. Consequently, 25% less language activations were excluded compared to all activations on the entire map. This led to the highlighting the LAs and the exclusion of unwanted activations and artifacts. The difference of 25% was the best possible result, therefore the general *t*-limit was set to *t* = 2.2.

### Filter parameters

**Figure 3:**
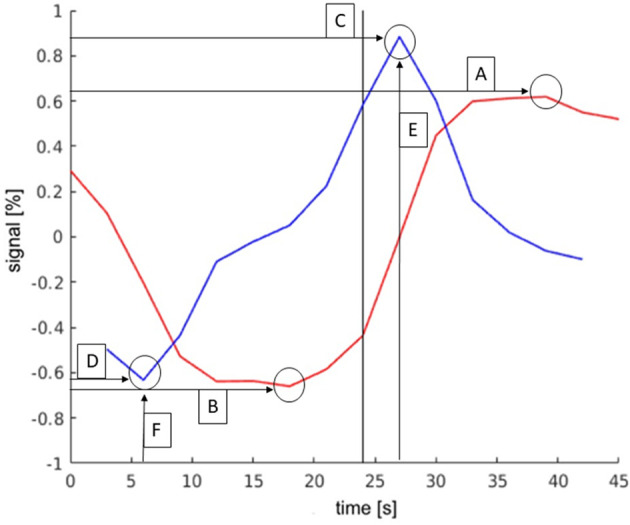
Exemplary TC and its derivation of language activations to demonstrate the parameters. The *y*-axis describes the deviation in the percent of the signal from the mean value of the signal. The black bar indicates the start of the activation block (at 24 s). The parameters were as follows: (**A**): maximum signal strength; (**B**): minimum signal strength; (**C**): maximum slope; (**D**): minimum slope; (**E**): time of activation onset; (**F**): time of activation end.

Six parameters were defined by comparing the TCs of activations in different regions and through analyzing the differences. With these parameters activations of the LAs could be distinguished from activations that did not belong to the LAs. These parameters are shown in Figure 3 and are explained in more detail below.

### Maximum and minimum signal strength

**Figure 4:**
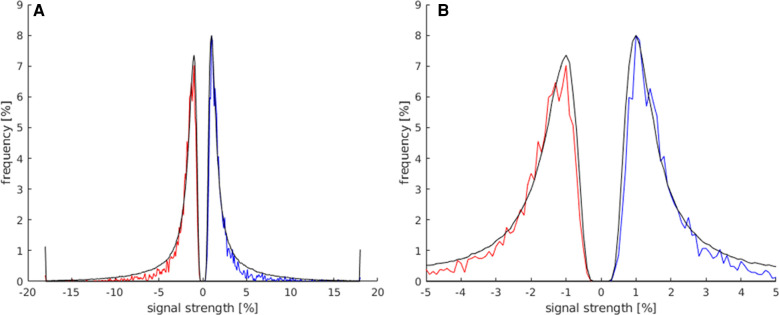
This histogram shows the comparison of the maximum and minimum signal strength values between all activated voxels of the t map (black) and the activated voxels in the LAs (red for minimum signal strength; blue for maximum signal strength). For this analysis, the data of all subjects were used. (**A**) This plot displays the frequency of voxels with signal strength values of −20%–20%. (**B**) Magnification of the plot in (**A**) (−5%–5% signal strength) to better illustrate the frequency distribution in the lower signal strength ranges.

the comparison between the signal strength maxima and minima of activations within the LA and the total number of all activations displayed on the whole map is presented in Figure 4. Both groups showed a frequency peak at approximately ±1%, and the calculated mean of the signal strength maximum was similar (both groups at approx. ±2%). These values were, therefore, not suitable for the identification of language activations. However, the number of activations in the LAs decreased faster from approx. ±3%, and beyond a signal strength value of approx. ±5.5%, a considerably smaller number of activations existed in the LAs compared to the total number of activations of the map. Also, the LAs showed fewer activations at signal strengths <±0.5%, compared to the total number of activations of the map.

The allowed limit for the maximum signal strength was set to 0.5%–5.5% and to −0.5% to −5.5% for minimum signal strength, deleting as few language area activations as possible and achieving the best results across all maps.

This parameter was used to identify clear apparent activations with an abnormally high or low max./min. signal strength. Sometimes voxels in the center of the language areas were still unselected, despite the widely set limit (±5.5%), suggesting either the existence of a large blood vessel or the center of activation. Overall, the use of this parameter led to an improvement of the *t*-maps.

#### Maximum and minimum slopes

**Figure 5:**
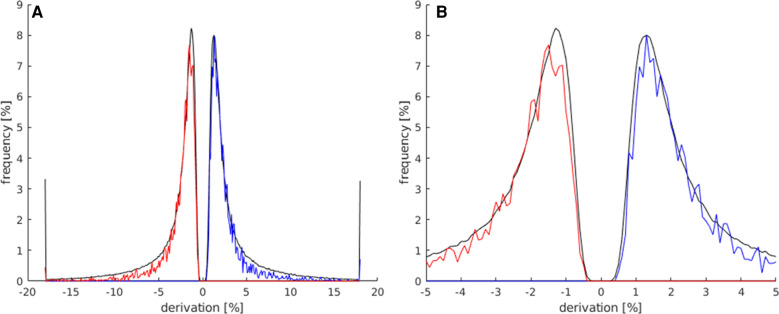
In this histogram, the maximum and minimum slope values of all activated voxels of the map (black) are compared to all activated voxels in the LAs (red for slope minimum; blue for slope maximum). For this analysis, all activation maps of the subject study were used. (**A**) This plot displays the frequency of activated voxels showing the slope maximum/minimum be-tween −20 and 20%/s. (**B**) Magnification of the plot in (**A**) (−5%/s–5%/s) to better illustrate the frequency distribution in the lower max./min. slope ranges.

The signal of the activations in the LAs was expected to rise quick and distinctly at the start of the activation block and decrease quickly after the end of the activation block. The activations in the LAs were, therefore, expected to show higher slope values. It is demonstrated in Figure 5 that activated voxels in the LA showed a max./min slope between ± and 3%/s more frequently than the rest of the activations.

The limit of this parameter was set to ±0.5%/s, to avoid the exclusion of too many language activations with a generally lower max./min slope. Below this limit, it became apparent that a quick and distinct rise and fall of the signal had not taken place, suggesting an activation independent from the stimulus. A few language activations were still excluded within this limit, but less compared to the excluded activations on the whole map.

As shown in Figure 5, the majority of activated voxels behaved similarly to the parameter signal strength at the higher slope maximums/minimums. The number of activations of the LAs decreased faster from approx. ±3%/s. At a max. slope of approx. ±4%/s and above, there were significantly less language activations compared to all activations on the map.

The allowed limits for the parameter minimum slope were set to 0.5%/s–5%/s for the pos. maximum and −0.5%/s to −5%/s. Using this parameter, many apparent not showing the LA-typical quick and distinct rise of the signal, followed by the decrease in the signal after the end of the activation, were identified and could be excluded from the maps.

#### Timing of maximum and minimum slope

The timing of the maximum slope represented the onset of activation or rather the time to peak. Based on the mechanism of the BOLD effect, a plateau of elevated signal was expected 6–8 s after the first presentation of a stimulus. In this experiment, the activation block started after timepoint 8, at second 24. The TCs of activations in the language areas were expected to rise between 24 s (onset of activation) and 30 s (full activation), as shown in Figure 3.

**Figure 6:**
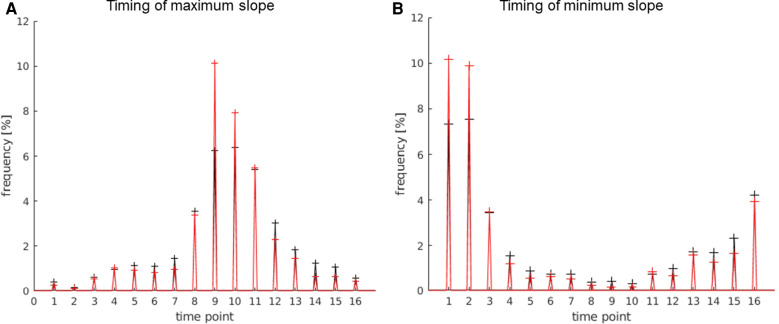
Timing of the max. slope (**A**) and min. slope (**B**); 1 timepoint = 3 s. The values of the language areas are displayed in red; the values of the total number of activations displayed on the map in black. (**A**) Many activations within the language area have a slope maximum between timepoint 8 and 12, i.e., 24 s and 36 s, with the highest number at time point 9, i.e., second 27, which is 3 s after the start of the activation block. (**B**) Most activations within the language area have the slope minimum between timepoint 16 and 3, i.e., 48 s and 9 s, with the highest number at time point 1, i.e., 3 s after the beginning of the rest block and after the end of the activation block.

The behavior of the HRF of activations in the LAs was as expected and is demonstrated in Figure 6: maximum rise in the signal at the beginning of the activation block; maximum decline in the signal at the beginning of the rest block (after the end of the activation block). The activations of other regions showed a similar, but not as prominent, pattern in the timing of the activation, which is obvious, since many regions outside the LAs were also activated by the stimulus used in the experiment. Thus, there were less activations showing the pos./neg. maximum directly at the start or end of the stimulus on the whole map compared to the LAs. Activations not starting at the beginning of the activation block were most likely independent from the presented stimulus and therefore counted as artifacts.

The allowed timing of the slope maximum was set to time point 8–12 (24 s–36 s), and the allowed timing of the slope minimum was set to timepoint 16 and 1–3 (45 s–48 s and 1 s–9 s).

Using this parameter, artifacts and unwanted activations were identified, improving the accuracy of the localization of the language areas.

### Application of combined parameters

**Figure 7:**
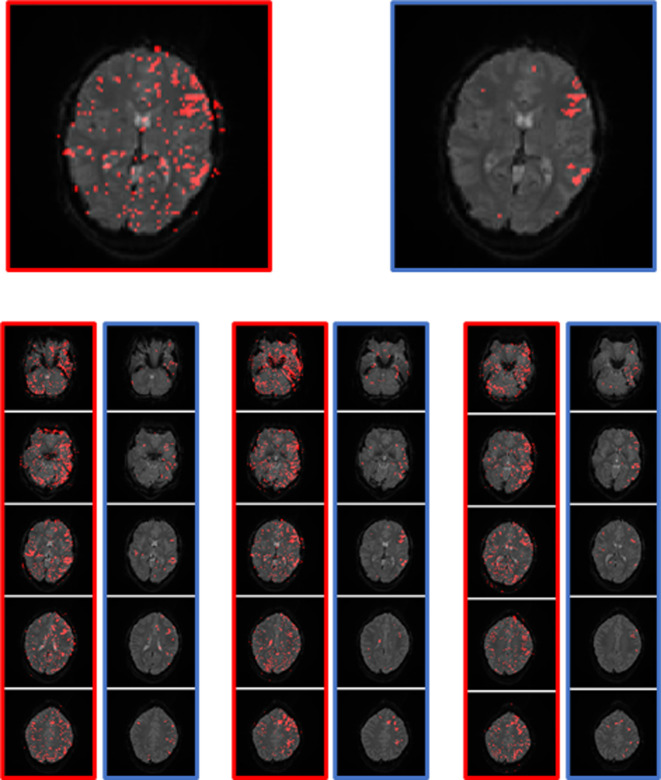
Example of the performance of the simple *t*-map (left, red) and the filtered *t*-map (right, blue). The two images on the top are the magnification of a slice with a good display of the language areas.

All parameters (Table 1) were combined and applied to the *t*-maps. The effect of the applied filter is shown in Figure 7, by comparing the filtered *t*-map to the simple *t*-map There were still a few artifacts and activations in other functional areas, directly stimulated by the tasks (visual cortex and motor areas used for language production (e.g., tongue movement)), displayed on the filter *t*-map. Thus, the filtered *t*-map provided an overall clearer display of language activations, as well as a strong reduction in artifacts.

**Table 1:** Limits for each parameter.

Parameter	Bottom limit	Top limit
Max. signal strength	0.5%	5.5%
Min. signal strength	−0.5%	−5.5%
Max. derivation	0.5%/s	6%/s
Min. derivation	−0.5%/s	−6%/s
Timing of max. slope	24 s	36 s
Timing of min. slope	48 s	9 s

The filtered *t*-map was created using a single voxel TC evaluation and the TCs of the single voxels were showing differences to some degree. This is the reason why the allowed limits of the parameters had to be set with leeway, so that as few voxels located in the LAs as possible were excluded. It was therefore unavoidable that some non-language activations with a similar TC were plotted as activations on the filtered *t*-map.

Of relevance is that all voxels displayed on the filtered *t*-map had a TC similar to language activations and were most likely activated by the experiment. Furthermore, many apparent activations were excluded, improving the appearance of the map, and making it easier to identify the LAs only by excluding apparently activated voxels, without changing the location of activations or adding activations.

Using the filter on the unsmoothed data enabled the detection of structural features, originating from gyri and sulci and veins. Especially the course of veins, which can sometimes be detected by the shape of activated areas, or deleted activations, is interesting for the evaluation of fMRI, since veins can lead to signal changes. The preservation of these structural features is mostly lost when applying smoothing, even though it can improve the localization of LAs.

### Performance of the filter

With the filter many artifacts and unwanted activations were removed from the raw fMRI *t*-maps, making the evaluation filter *t*-maps easier and providing easily distinguishable language activations and a realistic location of the language activations as they were measured.

Three aspects were considered rating the performance of the filtered *t*-maps compared to the preprocessed GLM maps used in the clinic: the display of the LA, the existence of directly adjacent activations that are not part of the LAs and the ratio between the targeted activations of the LAs and unwanted activations of other functional areas or artifacts.

An evaluation using only the subject data may be biased, since these data were used to analyze the TCs and develop the parameters and their limits. For this reason, the set of fMRI data that was obtained from the patient control group (*n* = 28) was used to verify the results.

The first part of the evaluation checked whether the LA were displayed on the activation maps. This was the simplest and most important part. Through this evaluation, it was possible to make a statement about the suitability of the filter *t*-map for the main objective of this examination: the display of the LAs. The comparison with the (through Siemens) verified clinically suitable GLM map is important, as in some cases, poor quality of the fMRI data or measurement errors make the evaluation and thus the display of the LAs impossible.

The results of this evaluation are shown in Table 2. Only minor differences between the two different mapping methods occurred, as both consistently displayed the language areas. It can be concluded that the filter *t*-map is suitable for the display of LAs and works as consistently as the evaluation program used in the clinic. The filter *t*-map achieved similar results to the GLM map, not only with the data from the subjects, but also with the independent data of the patient control group, suggesting that there is no bias and the method can be generally applied to fMRI data.

**Table 2:** This table describes how many activation maps displayed language activations (of Broca's and Wernicke's areas) in both maps.

Display of LAs	GLM map	Filter *t*-map
Broca (subjects)	100% (24)	100% (24)
Broca (patients)	96.4% (27)	100% (28)
Wernicke (subjects)	100% (24)	100% (24)
Wernicke (patients)	78.6% (22)	78.6% (22)

The display of Wernicke's area was equal in both. Wernicke's area was not displayed in a few protocols, which, due to the occurrence with both methods, should be attributed to the fMRI data and not to the evaluation method.

**Figure 8:**
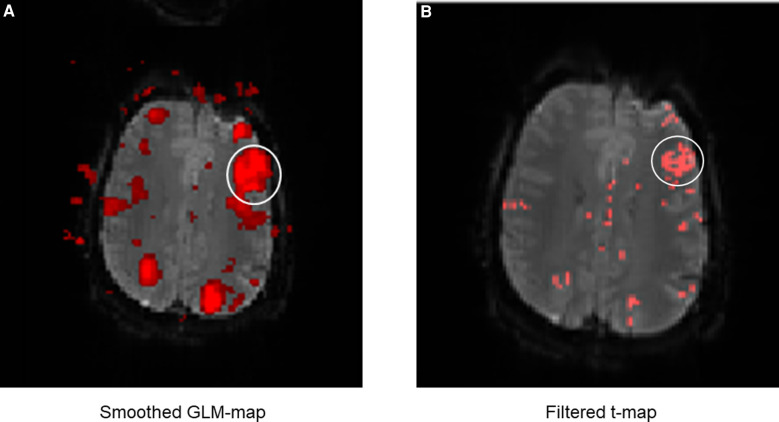
Exemplary comparison of the display of LAs between the GLM map (**A**) and the filter *t*-map (**B**). The white circle marks Broca's area. It can be noticed that the display of Broca's area is displayed much larger on the GLM map than on the filter *t*-map, even though the same fMRI data were used. This is mainly due to the smoothing of the GLM maps, causing surrounding activations to merge into the language activations (in this case of Broca's area). This can lead to differences between the displayed and truly measured spatial boundaries of the LAs. The presence of directly adjacent and merging activations complicates the evaluation and the exact localization of the LAs and was incorporated as a negative aspect in the evaluation of the activation maps. The display of Broca's area on the filter *t*-map (**B**) in comparison is smaller, distinguishable from sur-rounding activations and shows only the truly measured activations and spatial boundaries of activations.

The next part of the evaluation focused on the display and readability of the language activations. For the comparison of both methods, the display of language activations was evaluated depending on the presence of directly adjacent activations (Figure 8).

The results (Table 3) showed that activations directly adjacent to the language activations were displayed less frequently on filter *t*-maps than on GLM maps. Especially with the fMRI data of the subjects, which were mostly of high quality, the LAs could be displayed more clearly by using the filter *t*-map. When displaying Broca's area, only eight directly adjacent activations were found on the filter *t*-maps, while the GLM maps showed 13 directly adjacent activations. In a direct map-to-map comparison, the filter *t*-map displayed Broca's area better on 6 maps, while the GLM map was superior in only one case. On six maps, both methods showed directly adjacent activations to the LAs.

**Table 3:** Here, the frequency of the display of additional activations directly next to the LAs is compared among the methods.

Activations directly adjacent to the LAs	GLM map	Filter *t*-map
Broca (subjects)	54.2% (13)	33.3% (8)
Broca (patients)	82.1% (23)	57.1% (16)
Wernicke (subjects)	41.7% (10)	12.5% (3)
Wernicke (patients)	35.7% (10)	35.7% (10)

The filter *t*-maps also achieved good results with the display of Wernicke's area. While the GLM map showed activations directly adjacent to the LAs in ten protocols, only three were found on the filter *t*-map. In direct comparison, eight filter *t*-maps were better and one worse compared to the matching GLM map.

As before, the evaluation was performed in the patient control group (*n* = 28), since the subject data were used to analyze the TCs and develop the parameters and their limits. The results largely matched the evaluation of the subject data, with an improved display of Broca's area, but only an equal display of Wernicke's area of the filter *t*-map compared to the GLM map. There were 16 directly adjacent activations of Broca's area on the filter *t*-maps, while the GLM map showed 23. In direct comparison, eight filter *t*-maps were better and one worse compared to the matching GLM map.

In the display of Wernicke' area, the maps performed equally well, each displaying ten directly adjacent activations. In the direct comparison, both methods also performed equally.

The ratio between the number of activated voxels in Broca's and Wernicke's language areas and all other activated voxels was calculated in the third part of the evaluation of the mapping methods. A good activation map is characterized by a high number of activations in the targeted regions (here: LAs) and a low number of activations on the rest of the map. Some non-language activations occur frequently because the paradigms co-activate other regions (e.g., visual cortex); these co-activated regions are very difficult to exclude. Other regions and many artifacts are well detected by the filter or the preprocessing and the GLM and excluded. This comparison is intended to evaluate how well the LAs are highlighted in the filter *t*-map without preprocessing compared to the GLM maps used in clinical practice.

The ratios of the number of activated voxels in the LAs to all other activated voxels on the map at different cluster limits and thresholds are listed in Table 4, as well as the number of maps that did not show a language activation in a particular setting. For this analysis, patient and subject fMRI data were combined.

**Table 4:** Ratio of activated voxels in the language areas to all activated voxels on the map. Different *t*-limits and cluster limits were applied to show differences in the ratio. In addition, the number of maps that were excluded due to non-existing language activations is indicated. Fifty-two activation maps from both patients and subjects were used.

*t*-limit (threshold)	Cluster limit	Ratio GLM map (Brc–Wern)	Excluded maps (Brc–Wern)	Ratio filter *t*-map (Brc–Wern)	Excluded maps (Brc–Wern)
1.5	1	0.05	0.03	0	0	0.05	0.03	0	2
	5	0.05	0.04	3	4	0.08	0.08	5	8
2.0	1	0.06	0.04	0	1	0.05	0.03	0	2
	5	0.06	0.05	3	6	0.08	0.08	5	8
	9	0.07	0.05	4	6	0.11	0.12	11	15
	13	0.07	0.05	4	6	0.16	0.21	19	22
**2** **.** **2**	1	0.07	0.05	0	3	0.05	0.03	0	2
	**3**	**0** **.** **07**	**0** **.** **05**	**1**	**6**	**0** **.** **07**	**0** **.** **06**	**3**	**6**
	4	0.07	0.05	1	7	0.08	0.07	3	6
2.5	1	0.07	0.05	0	3	0.05	0.03	0	2
	5	0.08	0.06	3	7	0.08	0.08	5	8

Abbreviations: Brc, Broca's area; Wern, Wernicke's area.

The setting used in most of this work (cluster limit 3 and *t* = 2.2) showed a similar ratio of language activations to other activations in both methods, as well as the number of excluded data. In the GLM map and the *t*-map, activated voxels belonging to Broca's area accounted for 7% of all voxels; the ratio of activated voxels in Wernicke's area was 5% for the GLM map and 6% for the *t*-map.

Including the results of the various settings in the evaluation, the ratio of voxels per single language center (Broca's or Wernicke's area) tended to stay between 5% and 10%, resulting in high-quality activation maps, as seen before. Increasing the threshold led to slight improvements in the ratio between language and non-language activations in the GLM maps; on the filter *t*-maps, this had no effect, which was most likely due to the effect of the filter. The changes in the cluster limit had the opposite effect. While the increase in the cluster limit hardly had any effect on the GLM maps, due to the smoothing connecting most single voxels and small voxel groups, it led to drastic improvements in the ratio in the filter *t*-maps. However, it also led to a strong increase in excluded maps and was, therefore, limited as a method to improve the maps and highlight the LAs.

Overall, the ratio of language activations to non-language activations was quite similar for both maps, with the filter *t*-maps performing better overall due to the use of a small cluster limit. However, more maps were excluded using the filter *t*-maps, which was largely due to poor patient data. Many of those showed only very slight, incoherent activations, which were quickly excluded using the cluster limit. With the GLM maps, these activations could be displayed more coherently due to the smoothing and were, therefore, only excluded at higher cluster limits. While this may seem like an advantage of the GLM maps, it rather underpins the point that non-existent activations may be added to the maps by using excessive preprocessing, especially smoothing.

The comparison of the two mapping methods showed that the filter *t*-maps, created using a simple *t*-test and TC analysis, without smoothing or preprocessing, not only allowed a more realistic spatial representation of activations, but also led to an equally robust display of the LAs with an improved distinguishability of the LAs and an at least equal ratio between language activations and all other activation, highlighting and improving the identification of LAs.

## Discussion

The results showed that it was possible to create usable and high-quality activation maps with raw fMRI data, using a simple *t*-test and a TC-dependent filter. Compared to the GLM maps used in clinical routine, the filtered *t*-maps were of at least comparable quality, often with an improved display of the LAs.

By using raw, unprocessed fMRI data and avoiding smoothing, the localization of the displayed activations on the filter maps is the truly measured location and can be considered more accurate. Whether this is the true location of functional activity can only be clarified *via* intraoperative electro-cortical stimulation (ECS) (11).

Of course, the preprocessing of the data has a purpose in many investigations and situations and may sometimes be fundamental for the evaluation of the data, e.g., motion correction in the case of unsteady patients or very-high-resolution spatial measurements; normalization is important to be able to compare large patient collectives in neuroscience studies. Smoothing is also a very important tool in functional imaging and can improve and facilitate the evaluation of activation maps. However, it is important to keep the (clinical) question in mind and to compare the alternation of the data with the benefit that is expected from the preprocessing step.

This study focused on the preoperative mapping of the language areas. In this case, the spatial accuracy and authenticity of the displayed activations are important to identify the LAs correctly and to make valid predictions, enabling preoperative planning. The goal was to change the data as little as possible to display the activations of the LAs as realistically as possible.

The simplest way to create an activation map without changing the data is to use a *t*-map. However, basic *t*-maps contain many artifacts, and are not LA- or experiment-specific, as all temporal information is lost. For this reason, the TC characteristics of the LAs and the experiment were defined to create a filter that significantly improved the maps and the display of the LAs, without changing or adding to the measurement data. This also allowed the temporal data obtained from the setup of the experiment (start and end of the stimulus) to be incorporated into the identification of the LAs.

The used parameters have been described by different studies to identify interregional differences in the HRF (15–18, 20). Despite the existing interpersonal differences (14), mainly in the variety of signal and slope strength of the different patients and subjects, the right limits for the parameters were determined, and it was possible to improve the *t*-maps, only excluding, not changing or adding, activations.

The only way to create a more accurate display of the LAs was to exclude non-language activations, as the goal was to display only truly measured activations. This resulted in only true activations, but there was also a risk that too many activations were excluded, due to a too high *t*-value or cluster limit, or due to too narrow parameter limits.

The described evaluation method offered the possibility to easily implement additional parameters, which can lead to a further improvement in the creation and evaluation of the activation maps. The parameters can be designed to be question- and experiment-specific. The display of other functional brain areas and (most likely) higher cognitive areas could be improved with this method by adjusting the parameters and limits to the specific TC of the respective region.

Furthermore, the use of the HRF-dependent parameters gave the examiner more information about the data. For example, vein-dependent activations that were deleted from the filter due to their HRF could be recognized by their shape, providing important information about the signal in this area, as veins can change the HRF signal of surrounding activations (21, 22). This information was largely lost in the smoothed GLM maps. Also, the information of the course of gyri and sulci is lost in smoothed fMRI maps and the activations are displayed independently from anatomical boundaries (11). With the method presented here, the fine resolution of the activations is higher, and it can be expected that the course of anatomical boundaries (gyri and sulci) contributed to an improved display of the activations. This additional information about the source of the activations can lead to a more realistic localization of the language areas by trained experts.

The implementation of this experiment in a clinical setting and the verification of the performance of the filtered *t*-maps with data from the independent patient control group showed that this method can be transferred to clinical use cases. Particularly in the case of patients with impaired cognitive functions or neuroanatomical abnormalities due to previous neurosurgical interventions, tumors, or other processes leading to expanding lesions, multifaceted information is of great importance for a valid evaluation. This is shown by the improved representation of the language areas in the patients' activation maps.

The advantage of the filtered *t*-map over the smoothed GLM maps is that raw data, instead of preprocessed and smoothed data, are used and, accordingly, the truly measured location, which is most likely close to the real location of activations, is displayed on the activation map. The use of the filter, which included important information about the HRF in the creation of activation maps, enabled the creation of easily analyzable and artifact-poor results, showing the truly measured localization of activated voxels, without adding to or changing the data.

## Conclusion

It was demonstrated that maps, comparable to the current standard in terms of quality and evaluability, can be created by using unmodified, especially unsmoothed, data with a simple *t*-test and the use of HRF-dependent parameters. The advantage of this unmodified approach is that a more realistic estimate of the location of the language areas is provided as the activations are displayed as they were measured, without adding or changing the data. The use of the HRF parameters, beginning and end of the activations, slope of signal and signal strength led to the improved identification of LAs, as many apparent activations could be deleted, and voice activations merged less frequently with directly adjacent activations. In addition, an adjustment of the fMRI evaluation based on the experiment and indication is possible.

## Data Availability

The raw data supporting the conclusions of this article will be made available by the authors, without undue reservation.
